# UK–Africa Leadership Fellowship for Antimicrobial Stewardship (UK-ALF-A): A Bilateral Learning Approach to Advancing AMS Expertise Among Pharmacists

**DOI:** 10.3390/antibiotics15090833

**Published:** 2026-08-27

**Authors:** Siân Price, Yen Truong, Paria Sanaty Zadeh, Gloria Tumukunde, Claire Brandish, Edwin Panford-Quainoo, Sarah Cavanagh, Esther Obiri-Yeboah, Maxencia Nabiryo, Elizabeth Ward, Gizem Gülpinar, Victoria Rutter, Helena Rosado, Chikondi Savieli

**Affiliations:** Commonwealth Pharmacists Association, 66–68 East Smithfield, London E1W 1AW, UK; sian.price@commonwealthpharmacy.org (S.P.); yen.truong@commonwealthpharmacy.org (Y.T.); paria.sanatyzadeh@commonwealthpharmacy.org (P.S.Z.); gloria.tumukunde@commonwealthpharmacy.org (G.T.); claire.brandish@commonwealthpharmacy.org (C.B.); edwin.panfordquainoo@commonwealthpharmacy.org (E.P.-Q.); sarah.cavanagh@commonwealthpharmacy.org (S.C.); esther.obiriyeboah@commonwealthpharmacy.org (E.O.-Y.); maxencia.nabiryo@commonwealthpharmacy.org (M.N.); elizabeth.ward@commonwealthpharmacy.org (E.W.); gizem.gulpinar@commonwealthpharmacy.org (G.G.); victoria.rutter@commonwealthpharmacy.org (V.R.)

**Keywords:** antimicrobial stewardship, antimicrobial resistance, pharmacy leadership, advanced pharmacy practice, UK–Africa Leadership Fellowship, mixed-methods evaluation

## Abstract

**Background**: Antimicrobial resistance is a growing global health challenge, particularly in low- and middle-income countries where antimicrobial stewardship (AMS) implementation and leadership capacity remain limited. Pharmacists play a central role in AMS but often lack access to structured leadership development. To address this gap, the Commonwealth Pharmacists Association developed the UK–Africa Leadership Fellowship in Antimicrobial Stewardship (UK-ALF-A), a 12-month bidirectional leadership development programme combining online learning modules, live webinars, peer-learning sessions, dual mentorship from UK- and Africa-based mentors, and workplace-based AMS continuous quality improvement projects. UK-ALF-A was designed to strengthen leadership capability among pharmacists in the United Kingdom and sub-Saharan Africa. **Methods**: This study evaluated changes in leadership capability among pharmacists participating in the programme and explored how fellows applied these capabilities within AMS-related practice. A mixed-methods evaluation was conducted with 40 fellows (20 UK, 20 Africa), using pre- and post-programme self-assessments and 360-degree colleague feedback based on the International Pharmaceutical Federation Global Advanced Development Framework (FIP GADF), alongside post-fellowship surveys and semi-structured interviews. **Results**: The proportion of fellows self-assessing at the highest competency level increased from 10% pre-programme to 39% post-programme, with corresponding increases in colleague feedback ratings from 17% to 49%. Qualitative data from open-ended survey responses and semi-structured interviews suggested improvements in leadership confidence, communication, and ability to influence AMS practice. Fellows reported leading prescribing improvements and multidisciplinary engagement, often without formal authority, with greater relative gains observed among Africa-based participants. **Conclusions**: UK-ALF-A was associated with enhanced leadership capability and contributions to AMS practice, alongside reported changes in individual and organisational practices that support broader health system strengthening, highlighting its value as a workforce development model.

## 1. Introduction

### 1.1. Leadership Development in Antimicrobial Stewardship

Antimicrobial stewardship (AMS) is critical for optimising antimicrobial use, improving patient outcomes, and mitigating antimicrobial resistance (AMR), a growing global threat affecting humans, animals, and the environment. Despite international prioritisation of AMR, many health systems, particularly in low- and middle-income countries (LMICs), face challenges in implementing sustainable AMS programmes. These challenges include limited workforce capacity, infrastructure constraints, and insufficient leadership capability [1].

Pharmacists play a central role in AMS delivery, requiring both technical expertise and leadership capability, and are increasingly expected to influence prescribing practices, lead quality improvement initiatives, and drive system-level change. Leadership is recognised as a core competency in pharmacy practice frameworks, including the International Pharmaceutical Federation (FIP) Global Advanced Development Framework (GADF) [2] and UK leadership models [3,4]. These frameworks highlight the importance of skills such as collaboration, influencing, data collection and analysis, change management and system-level thinking.

However, access to structured leadership development opportunities remains limited in many LMIC settings [1]. Pharmacy education and training has traditionally prioritised technical competencies, with fewer opportunities for mentorship, strategic leadership development, experiential learning or system-level engagement. As a result, pharmacists may be well positioned to support AMS but lack the confidence, capability, or authority to lead change effectively.

Strengthening leadership capability among pharmacists therefore represents an important and underexplored component of global AMS capacity building. Leadership development experiences within health partnerships provide a practical and scalable approach to building sustainable workforce capability while supporting organisational improvement and broader health system strengthening.

### 1.2. Development of Leadership Fellowship Programmes

Recognising the need for leadership development within the pharmacy workforce, fellowship models have been developed within global health partnership settings. The Chief Pharmaceutical Officer’s Global Health Fellowship (CPhOGHF) for UK-based pharmacists introduced experiential leadership development within global health partnerships and demonstrated improvements in leadership capability and service delivery outcomes [5,6]. The programme was developed by the Commonwealth Pharmacists Association (CPA) and delivered through the Commonwealth Partnerships for Antimicrobial Stewardship (CwPAMS), a health partnership programme supporting AMS capacity strengthening across sub-Saharan Africa [7,8].

Subsequent scoping work undertaken by the CPA identified significant unmet leadership development needs among pharmacists in Africa, including gaps in strategic thinking, behaviour change, project management, advocacy and influencing skills [1]. In response, the African Leadership Fellowship in AMS (ALF-A) was developed and delivered between October 2023 and January 2025 [9].

Building on lessons from both programmes, the UK–Africa Leadership Fellowship in AMS (UK-ALF-A) was established as a bidirectional leadership development programme bringing together pharmacists from the UK and sub-Saharan Africa. The fellowship was designed to promote shared learning, cross-cultural collaboration, and leadership development within the context of AMS improvement.

### 1.3. The UK–-Africa Leadership Fellowship in Antimicrobial Stewardship

UK-ALF-A was a 12-month fellowship developed by CPA and delivered between April 2025 and March 2026 within CwPAMS. The programme brought together early- to mid-career pharmacists from the UK and seven sub-Saharan African countries in a single cohort, and combined structured leadership and AMS learning with applied workplace practice, including:

- Online learning modules;
- Live webinars and peer-learning sessions;
- Dual mentorship from UK- and Africa-based mentors;
- Delivery of AMS-focused continuous quality improvement (cQI) projects.

The curriculum covered global health, AMS, leadership, behaviour change, and project management, drawing on internationally validated frameworks from the World Health Organisation [10] and FIP [2]. Fellows applied their learning through workplace-based AMS cQI projects aligned with national, regional, local and CwPAMS partnership priorities. The fellowship was informed by experiential and workplace-based learning principles, whereby leadership capabilities are developed through structured learning, mentorship, reflection and application to real-world AMS challenges.

Mentorship was provided through a dual model, with each fellow supported by both a UK-based and an Africa-based mentor. In addition, an Alumni Mentoring Programme engaged previous CPhOGHF and ALF-A fellows as trained mentors to support participants, contributing to programme sustainability and leadership pipeline development.

The fellowship included participants from the UK and seven sub-Saharan African countries. Its integrated design aligned individual leadership development with partnership objectives and broader AMS system-strengthening.

This study aimed to evaluate the impact of UK-ALF-A on leadership capability development and its contribution to AMS practice.

## 2. Results

### 2.1. Participant Demographics

A total of 40 fellows took part in the programme: 20 from the UK and 20 from sub-Saharan African countries (Ghana, Kenya, Malawi, Nigeria, Tanzania, Uganda and Zambia). Participant demographic characteristics are summarised in Table 1. Fellows differed in gender distribution, academic qualifications and baseline career characteristics, although the cohort was broadly representative of early- to mid-career pharmacists across both regions. Since the evaluation included the entire fellowship cohort and was not designed to compare groups statistically, demographic data are presented descriptively only.

Overall, the cohort was predominantly female (75%), with a greater proportion of female participants in the UK (90%) than in Africa (60%). Most fellows were aged 31–40 years (52.5%) and had between 6 and 15 years of pharmacy practice experience (65.0%). All UK-based fellows held an MPharm qualification, whereas Africa-based fellows had more varied educational backgrounds, most commonly a Bachelor’s degree in pharmacy (75%).

### 2.2. Pre- and Post-Fellowship Assessments

#### Self-Assessment and 360-Degree Feedback

All 40 fellows completed the pre-fellowship self-assessment and 37 fellows completed the post-fellowship self-assessment. There were 173 pre-fellowship and 115 post-fellowship responses from fellow colleagues.

**Figure 1:**
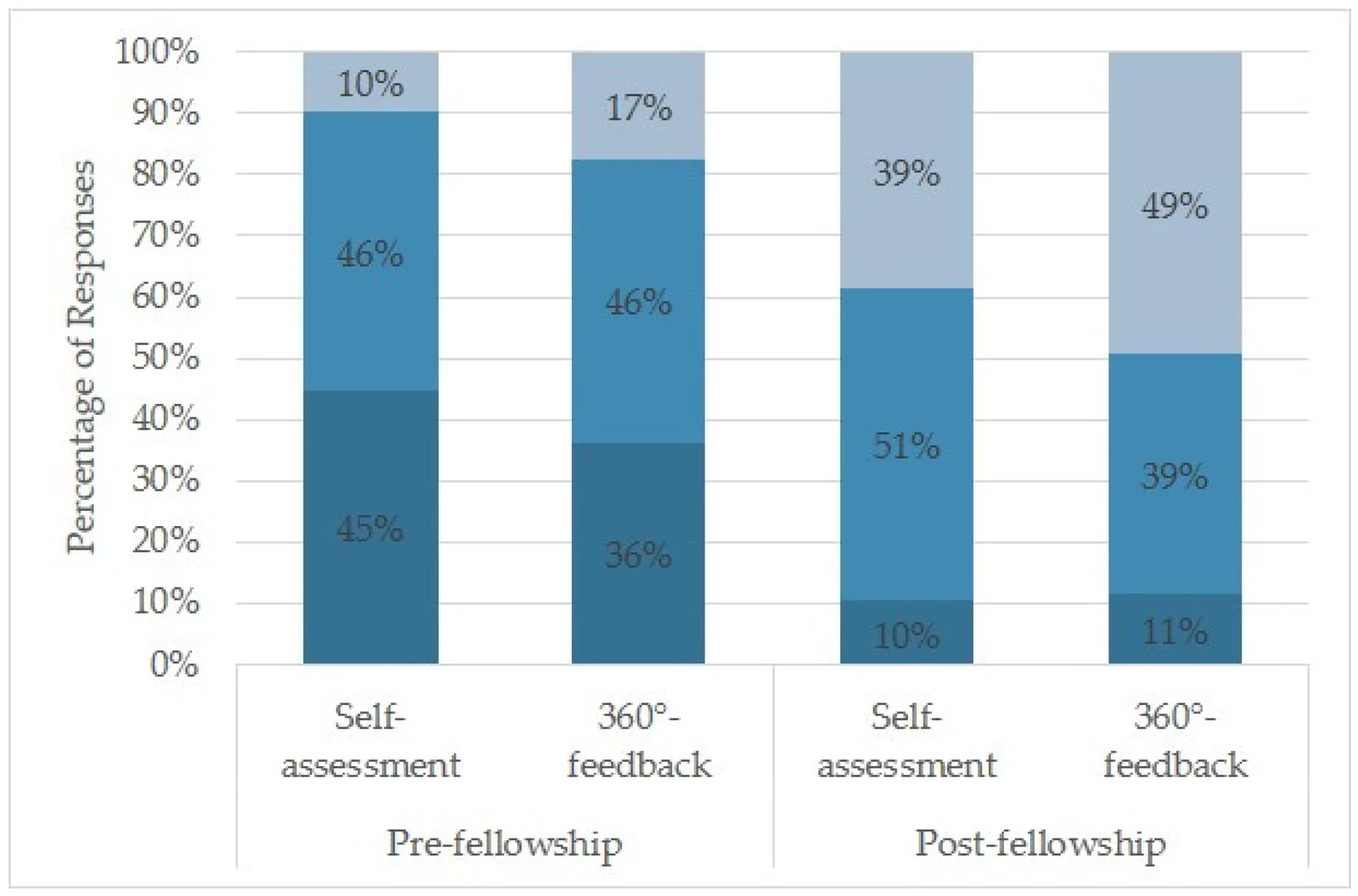
Overall shift in advanced practice competency dimensions (*n* = 4 Clusters Aggregated). Combined 100% stacked bar charts display the total distribution of responses across all four FIP GADF clusters, comparing Fellows’ self-assessments (*n* = 40 pre-fellowship, *n* = 37 post-fellowship) against 360-degree colleague feedback (*n* = 173 pre-fellowship, *n* = 115 post-fellowship) pre- and post-fellowship training. Dark blue segments (▮) represent Advanced Stage 1, medium blue (▮) represents Advanced Stage 2, and light blue (▮) represents Advanced Stage 3. Note the overall proportional growth into Advanced Stage 3 following fellowship completion.

**Figure 2:**
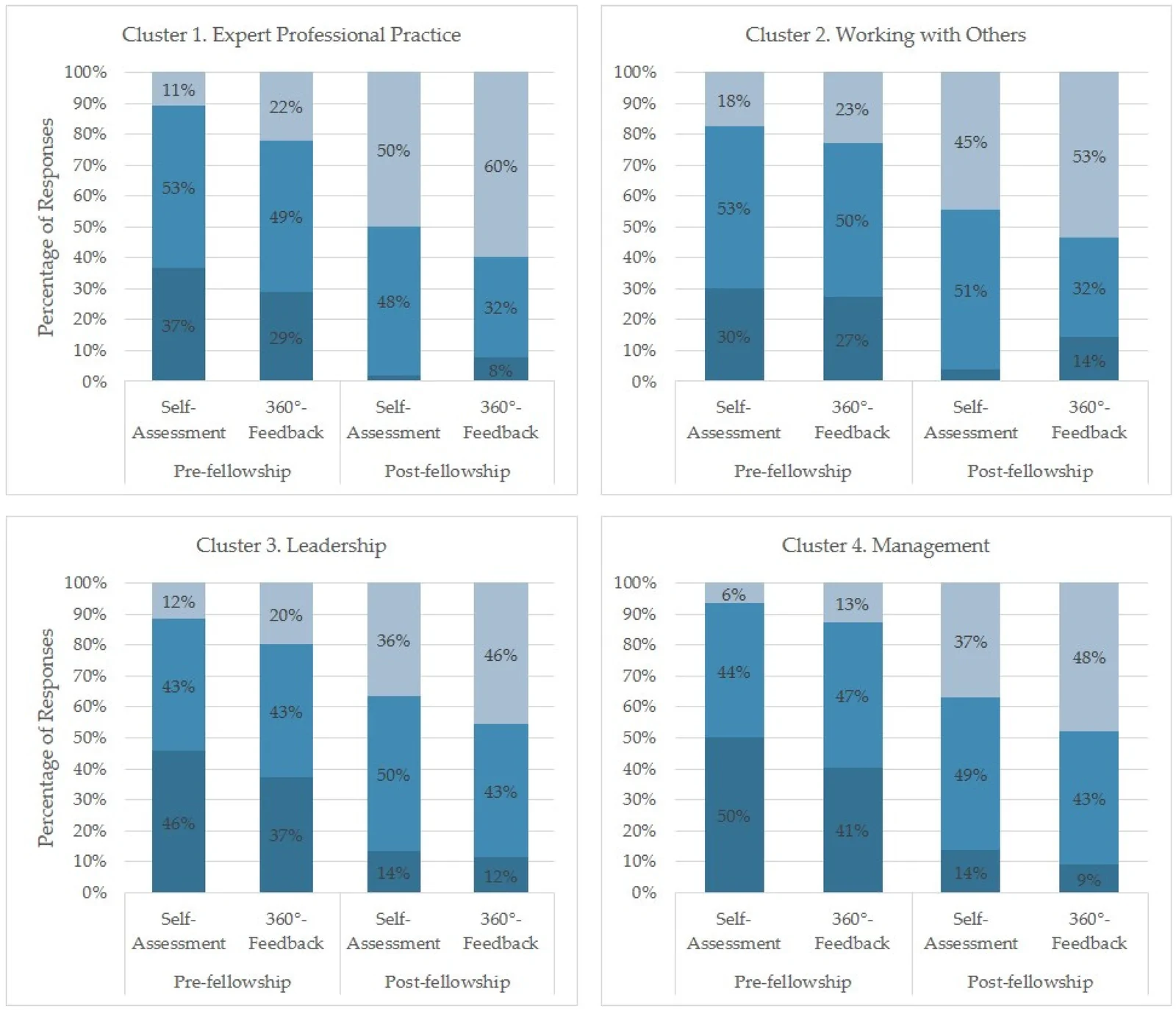
Distribution of advanced practice competency stages pre- and post-fellowship. Multi-panel 100% stacked bar charts display response distributions across four framework domains: Cluster 1 (Expert Professional Practice), Cluster 2 (Working with Others), Cluster 3 (Leadership), and Cluster 4 (Management). Within each cluster, data compare Fellows’ self-assessments (*n* = 40 pre-fellowship, *n* = 37 post-fellowship) against multi-source 360-degree colleague feedback (*n* = 173 pre-fellowship, *n* = 115 post-fellowship). Shading denotes competency progression: dark blue (▮) represents Advanced Stage 1, medium blue (▮) represents Advanced Stage 2, and light blue (▮) represents Advanced Stage 3. Note the proportional growth into Advanced Stage 3 for most clusters following fellowship completion.

Fellows’ self-assessments showed a substantial shift toward higher competence stages following completion of the fellowship (Figure 1). Before the programme, most Fellows positioned themselves in Advanced Stage 1 (45%) or Advanced Stage 2 (46%), with only a small proportion (10%) identifying at Advanced Stage 3. This pattern was consistent across all four clusters of the FIP GADF (Figure 2). Post-fellowship ratings demonstrated a marked redistribution: the proportion of fellows reporting Advanced Stage 1 competence fell sharply to 10%, while those identifying in Advanced Stage 3 increased approximately four-fold to 39%. Advanced Stage 2 remained relatively stable (51%). Across every cluster, the proportion of participants rating themselves at the highest competence level increased substantially.

Colleagues’ 360-degree feedback suggested a similar pattern, with a strong upward shift in perceived competence across all clusters following the fellowship. Pre-fellowship ratings were as follows: Advanced Stage 1 (36%), Advanced Stage 2 (46%) and Advanced Stage 3 (17%). Post-fellowship assessments showed a substantial redistribution, with Advanced Stage 1 falling sharply to 11% and Advanced Stage 2 decreasing slightly to 39%, reflecting movement into the highest competence level. The overall proportion of ratings at Advanced Stage 3 increased from 17% to 49%, indicating that half of all colleague assessments placed fellows at the most advanced stage after completing the programme. All four FIP GADF clusters and overall 21-competency scores showed statistically significant improvements, with large positive rank-biserial correlations (Wilcoxon signed-rank *p* < 0.001, Table 2 and Supplementary Data).

Across all four FIP GADF clusters, clear differences emerged between self-assessments and 360-degree feedback both before and after the fellowship. Pre-fellowship, colleagues consistently rated fellows at higher competence levels than the fellows rated themselves, with a greater proportion of 360-degree feedback falling into Advanced Stage 3 across every cluster and fewer ratings in Advanced Stage 1. Following the fellowship, however, a strong convergence between the two assessment methods emerged. Self-ratings showed substantial upward movement, with Advanced Stage 3 increasing from 10% to 39%, mirroring the shift observed in colleague feedback, which rose from 17% to 49%. At the same time, both assessment types demonstrated sharp reductions in Advanced Stage 1 ratings (Figure 1).

Across all four FIP GADF clusters, both self-assessments and 360-degree feedback showed consistent increases in average competence stage ratings (1–3) following the fellowship, indicating meaningful development in advanced practice competence. The largest median self-assessment changes were observed in Expert Professional Practice (+0.75) and Leadership (+0.67). Corresponding improvements were observed in 360-degree feedback across all four clusters (all *p* < 0.001), with median changes ranging from +0.49 to +0.58 (Figure 2, Table 2).

**Figure 3:**
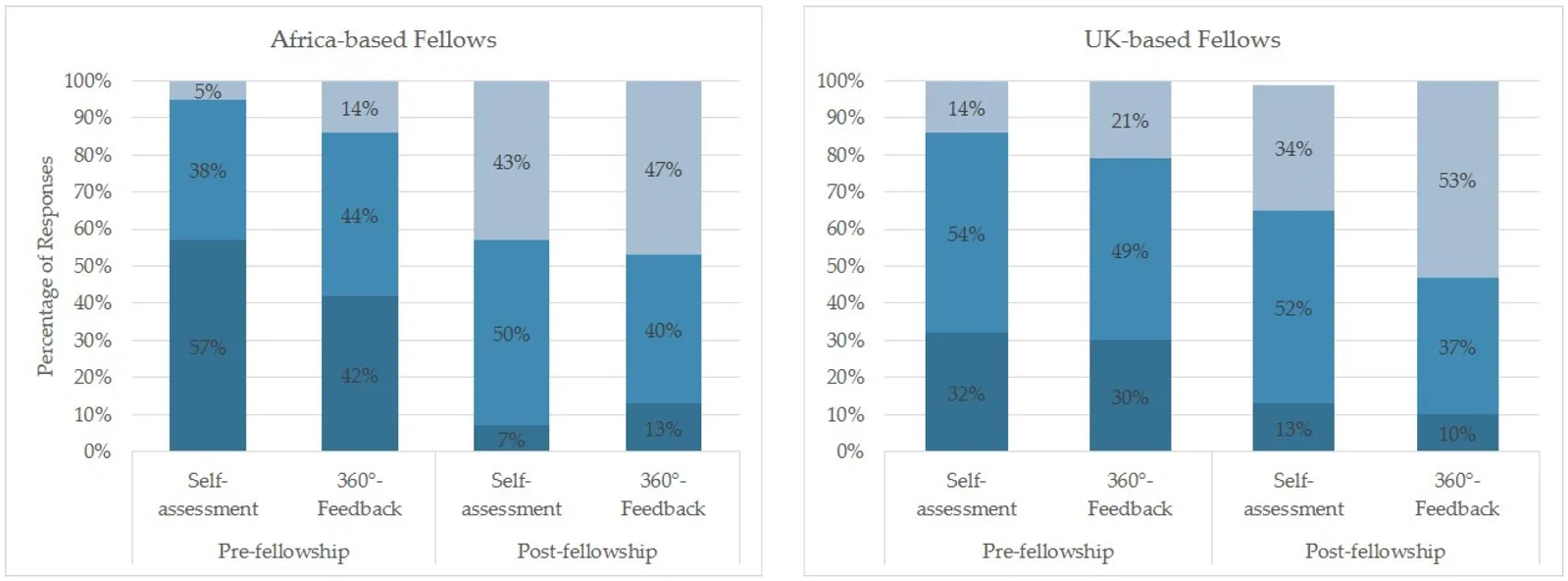
Regional differences in distribution of competence stages pre- and post-fellowship: ▮ Advanced Stage 1, ▮ Advanced Stage 2 and ▮ Advanced Stage 3. Percentages reflect proportion of total responses. Left: Self-assessment by Africa-based Fellows pre- (*n* = 20) and post- (*n* = 18) fellowship; 360-degree feedback by colleagues of Africa-based Fellows pre- (*n* = 89) and post- (*n* = 69) fellowship. Right: Self-assessment by UK-based Fellows pre- (*n* = 20) and post- (*n* = 19) fellowship; 360-degree feedback by colleagues of UK-based Fellows pre- (*n* = 84) and post- (*n* = 46) fellowship.

Across both Africa and the UK, the fellowship programme was associated with substantial upward shifts in competence, although the baseline levels and scale of improvement differed notably between Africa-based and UK-based fellows. Prior to the fellowship, Africa-based fellows were concentrated overwhelmingly at Advanced Stage 1 (57%), whereas UK-based fellows predominantly identified at Advanced Stage 2 (54%). Following the fellowship, self-assessments showed marked progression in both groups, with Africa-based fellows demonstrating the most pronounced transformation: Stage 1 ratings dropped from 57% to 7%, while Stage 3 rose sharply from 5% to 43%. UK-based fellows also improved, though from a higher starting point; Stage 3 increased from 14% to 34%. These trends were corroborated by 360-degree colleague assessments. Among Africa-based fellows, Stage 3 ratings from 360-degree feedback rose from 14% to 47%, with Stage 1 falling from 42% to 13%. UK-based fellows also showed a shift toward higher competence, with Stage 3 increasing from 21% to 53% and Stage 1 declining from 30% to 10%. Overall, while gains were observed across all FIP GADF clusters and both regions, the magnitude of advancement was greatest among Africa-based fellows (Figure 3).

### 2.3. Qualitative Findings

Thematic analysis of open-ended survey responses and semi-structured interview data identified consistent and overlapping themes relating to leadership development, organisational impact, mentorship, and programme experience. Across both data sources, findings demonstrated substantial professional and behavioural change, alongside practical challenges associated with programme delivery.

#### 2.3.1. Leadership Development and Behavioural Change in LMIC Health Systems

Developing AMS capacity within LMIC settings introduces operational challenges tied to organisational hierarchies, clinical power dynamics, and constrained resources. Traditional leadership approaches often fail to address the complex power structures that characterise multidisciplinary healthcare teams in LMICs. As highlighted by Aivalli et al. (2025) and MacKechnie et al. (2022), leadership interventions in LMICs require adaptive mechanisms that foster relational leadership and cross-sectoral negotiation [11,12]. Combining structured cQI projects with dual mentorship and peer learning as part of UK-ALF-A provided fellows with structured authority to challenge established antimicrobial prescribing patterns and navigate workplace hierarchies. This framing highlights leadership capability as an adaptive mechanism enabling pharmacists to exercise agency, implement stewardship protocols, and drive multidisciplinary engagement despite structural constraints.

Fellows consistently reported significant improvements in leadership confidence, self-awareness, and communication. These were often described as a shift in leadership identity, moving from task-focused or hesitant practice toward more confident, reflective, and adaptive leadership approaches. Participants highlighted greater willingness to contribute in multidisciplinary settings, engage in decision-making processes, and challenge established practices where appropriate: “*Before, I would attend meetings and maybe have opinions, but I wouldn’t speak out… now I feel more confident to contribute and challenge where needed.*” This transition was frequently linked to increased use of structured leadership approaches, including cQI methodologies, behaviour change principles, and reflective practice. Fellows described becoming more intentional in their leadership style, with greater emphasis on listening, understanding team dynamics, and adapting their approach to different individuals and contexts.

#### 2.3.2. Organisational and Wider AMS Impact

Fellows reported a meaningful impact on AMS practice and wider organisational processes. Common examples included improvements in antimicrobial prescribing and audit practices, strengthened multidisciplinary collaboration, and increased engagement in stewardship activities. In several cases, fellows described contributing to changes in local guidelines, expanding participation in committees, and leading education or training initiatives.

Importantly, these outcomes were often achieved without formal authority, relying instead on enhanced communication, stakeholder engagement, and influence. Fellows described increased confidence in engaging with prescribers and senior colleagues, and in facilitating discussions around antimicrobial use:

“*I now engage prescribers in evidence-based discussions and feel more confident to challenge when needed.*”

Some fellows also reported influencing resource allocation and strategic decision-making related to AMR and AMS, including contributing to discussions around budget priorities and shaping regional and national initiatives. These examples suggest that the impact of the fellowship extended beyond individual practice and local services to broader organisational and health-system levels.

These findings highlight the role of influence-based leadership in enabling AMS improvement within complex healthcare systems. They also demonstrate how leadership development can support individuals to shape policy, priorities, and stewardship activities even in the absence of formal managerial authority.

#### 2.3.3. Mentorship and Peer Learning

Mentorship was identified as a key enabler of leadership development across both datasets. Fellows valued mentors who provided guidance, encouragement, and context-specific insight, particularly in navigating organisational challenges and progressing AMS projects. In some cases, mentorship provided important psychosocial support, reinforcing confidence and resilience.

However, experiences were variable. Some fellows reported challenges with mentor availability, alignment of expertise, or the structure of mentoring sessions. There was a preference for more flexible, needs-based mentoring approaches, alongside clearer expectations at programme outset.

Peer learning and networking were also highlighted as important components of the fellowship. Opportunities to engage with colleagues across different countries and practice settings were valued for knowledge exchange, perspective-sharing, and professional support.

#### 2.3.4. Programme Experience, Challenges, and Accessibility

Despite high overall satisfaction, fellows consistently identified challenges related to programme delivery. Workload intensity was the most frequently reported issue, with many describing difficulties balancing fellowship requirements alongside full-time clinical roles. Participants often relied on personal time, time-management strategies, and mentor support to remain engaged.

Additional challenges included the timing and sequencing of programme components, particularly where leadership or cQI content followed the initiation of projects. Some fellows reported duplication across learning activities or variable relevance of certain components to their specific practice settings. Accessibility concerns were also identified, including limitations related to timing, digital delivery, and engagement with interactive sessions.

#### 2.3.5. Future Programme Improvement

Fellows suggested several areas for improvement to enhance future iterations of the programme. These included increasing opportunities for in-person or experiential learning, expanding hands-on AMS exposure (particularly in clinical environments), and strengthening research and publication support. Participants also highlighted the importance of clearer expectations, improved pacing of activities, and more consistent mentor engagement.

While some differences were observed between UK- and Africa-based fellows, particularly in relation to priorities for practical experience versus networking and research support, there was strong alignment across both groups in valuing experiential learning and improved programme coordination.

## 3. Discussion

This study provides mixed-methods evidence that UK-ALF-A is an effective model for developing AMS leadership capability and strengthening AMS practice across diverse health system contexts.

Substantial improvements were observed, including a nearly four-fold increase in fellows self-assessing at Advanced Stage 3 (10% to 39%), alongside similar gains in 360-degree feedback (17% to 49%). However, the key contribution of these findings extends beyond the magnitude of change, highlighting the role of structured, experiential, and mentored leadership development as a mechanism for strengthening AMS capacity. These findings should be interpreted within implementation science frameworks, which emphasise the importance of evaluating both outcomes and mechanisms of change in complex interventions [13]. Moreover, learning from UK-ALF-A should be interpreted as part of a broader evidence base on workforce development interventions that combine experiential learning, mentorship, and workplace-based quality improvement to drive behavioural change in healthcare professionals [14,15].

This programme evaluation builds on earlier CPA-led work, including the scoping study of leadership development needs in Africa [1], which identified systemic gaps in access to structured training, mentorship, and career pathways. Priority needs include strategic thinking, influencing skills, behaviour change, and system-level leadership. In response, ALF-A was developed as a context-specific programme for African LMIC settings and demonstrated that structured, relevant leadership training can improve confidence, communication, and engagement in AMS [9]. The current study extends this evidence by demonstrating not only improvements in self-assessed leadership capability but also corresponding shifts in colleague-rated practice competence and qualitative evidence of fellows applying leadership approaches in practice.

UK-ALF-A builds on this model in two key ways. First, it embeds leadership development within practical AMS implementation through fellows’ involvement in cQI-driven improvement projects, ensuring direct application to service delivery. This aligns with established quality improvement approaches in healthcare that emphasise iterative testing of change in real-world settings to improve care processes and outcomes [16,17]. Second, it adopts a bidirectional model, bringing together UK- and Africa-based pharmacists to enable reciprocal learning and deeper understanding of leadership across different health systems. This reflects growing recognition in global health of the value of equitable partnerships and knowledge exchange rather than unidirectional capacity building [7,18].

Together, these programmes form a coherent evidence base demonstrating that structured, mentored, practice-based fellowships can address leadership gaps and improve both professional competence and AMS delivery. The observed changes are consistent with experiential learning theory, which emphasises learning through cycles of action and reflection in real-world contexts. This is particularly relevant in professional development settings [9,19], and with contemporary workplace learning research highlighting the importance of situated learning in professional communities [20].

A central contribution of this study is the clear link between AMS-focused leadership development and AMS practice outcomes. Improvements across all FIP GADF domains, together with concordant findings from self-assessment and 360-degree feedback, provide convergent evidence of perceived progression in advanced practice competence. The qualitative findings further support this, with fellows describing shifts towards more confident, adaptive, and relational leadership, alongside increased ability to influence multidisciplinary teams and challenge prescribing practices. These findings are consistent with contemporary leadership theory in healthcare, which emphasises leadership as a relational and socially constructed process rather than a positional attribute [21,22,23]. Leadership development should be understood not as an abstract competency, but as a practical mechanism for enabling stewardship within complex clinical systems. The findings do not suggest that leadership development alone improves AMS outcomes; rather, leadership capability appears to act as an enabling mechanism through which pharmacists can influence prescribing practices, multidisciplinary collaboration and stewardship implementation.

The programme’s impact appears to have been driven by several interacting mechanisms. Experiential learning through cQI projects enabled fellows to apply leadership behaviours in real-world contexts, reinforcing learning through practice and reflection. Structured mentorship provided guidance and psychosocial support. This is widely recognised as a key enabler of professional development and identity formation in healthcare training environments [24,25] and provides a framework for translating learning into action. Peer learning across countries and settings further reinforced reflective practice and broadened understanding of leadership across different health system constraints, consistent with evidence on collaborative learning in interprofessional and global health education [26,27]. Collectively, these components likely contributed to the observed shift in leadership identity, moving from task-focused pharmacy practice toward more systems-oriented and adaptive leadership approaches.

UK-ALF-A also highlighted global inequities in leadership capacity. Africa-based fellows began at lower baseline levels but demonstrated the greatest gains, suggesting that structured leadership development interventions may be particularly impactful in settings where formal leadership training opportunities are limited [1]. This aligns with broader global health workforce evidence highlighting unequal access to professional development and the importance of targeted capacity-strengthening initiatives [14,15]. UK-based fellows also progressed, particularly in relation to system leadership and influencing practices. The bidirectional design therefore represents a key innovation, supporting mutual learning rather than unidirectional capacity building. This aligns with wider global health discourse emphasising equitable partnerships and shared learning models rather than traditional donor–recipient training structures [7,9,18].

Leadership impact was largely achieved through influence rather than formal authority. Fellows reported improvements in communication, stakeholder engagement, and multidisciplinary collaboration, enabling change within complex systems despite limited positional power. This is particularly important for AMS, where pharmacists often operate without direct decision-making authority in relation to prescribing, and consistent with distributed leadership theory, which emphasises that leadership is enacted across networks of individuals rather than residing solely in formal roles [28]. It also aligns with more contemporary views of adaptive leadership in complex healthcare systems, where influence, negotiation, and relational skills are essential for change [29]. The findings, therefore, reinforce the importance of relational and adaptive leadership skills in enabling stewardship outcomes, particularly in resource-constrained or hierarchical environments.

This evaluation adds to the relatively limited evidence on leadership development for pharmacists working in AMS by demonstrating measurable changes in advanced practice competency alongside qualitative evidence of increased confidence, influence and application of leadership approaches. The consistency between self-assessment and colleague-rated competence is encouraging, although both remain perception-based measures and should not be interpreted as objective evidence of behavioural or service-level change.

The findings support a view of leadership development as an important component of AMS workforce capacity building. UK-ALF-A was designed to develop capabilities such as influencing, communication, multidisciplinary collaboration, systems thinking and quality improvement, which are relevant to pharmacists working within complex AMS environments. Whether development of these capabilities translates into sustained changes in antimicrobial prescribing, stewardship implementation or patient outcomes remains an important question for future longitudinal evaluation.

While this study provides valuable insights into the impact of UK-ALF-A, several limitations should be considered when interpreting the findings. The evaluation cohort was relatively small (*n* = 40), reflecting the full programme enrolment, with fellows selected through a competitive recruitment process, which may limit generalisability and introduce selection bias. In addition, not all fellows and colleagues completed competency assessments, creating the potential for response and attrition bias. The quantitative outcomes were based on self-assessment and colleague-rated competency assessments, which remain subjective and may be influenced by social desirability or changes in familiarity with the FIP GADF framework. Although colleagues were asked to complete assessments at both time points, 360-degree responses were anonymous and individual respondents could not be linked longitudinally; therefore, changes reflect fellow-level aggregated colleague perceptions rather than matched respondent-level change. The pre-post design lacked a control or comparison group, so observed changes cannot be attributed solely to the fellowship. Finally, the evaluation did not measure objective downstream AMS outcomes such as antimicrobial consumption, prescribing appropriateness, guideline adherence, resistance indicators or patient outcomes. The findings therefore relate primarily to leadership capability and advanced practice competence rather than demonstrable changes in AMS outcomes.

Nevertheless, the consistency across self-assessment, colleague feedback, and qualitative findings strengthens confidence in the observed effects. The convergence of findings across self-assessment, colleague feedback and qualitative findings provides complementary evidence of changes in perceived advanced practice and leadership capability, while the limitations above should be considered when interpreting the magnitude and attribution of these changes. This triangulation strengthens internal validity and is consistent with established mixed-methods evaluation principles [30].

Overall, UK-ALF-A suggests that leadership development is not peripheral but central to AMS strengthening, offering an evidence-based model for workforce development globally. By combining experiential learning, mentorship, and bidirectional global partnerships, the programme provides a framework for developing adaptive leadership capacity capable of driving sustainable improvements in AMS and wider health system performance.

## 4. Materials and Methods

Forty pharmacists (20 from sub-Saharan Africa and 20 from the UK)—referred to as fellows—were selected to take part in the fellowship and enrolled in the UK-ALF-A between April 2025 and March 2026. Fellows were selected through a competitive recruitment process, based on demonstrated leadership potential, commitment to advancing AMS and evidence of institutional support. Fellows were early- to mid-career pharmacists working in a range of practice settings in the UK and Africa and linked to a CwPAMS Health Partnership.

UK-ALF-A was delivered through a hybrid model combining online learning, mentoring, and an in-person residential workshop. The virtual component included leadership development modules, peer-learning circles, and mentorship sessions. The programme was designed so that it was flexible enough to enable pharmacists working in any setting of practice to take part.

The evaluation used a mixed-methods approach, combining quantitative and qualitative methods to evaluate the programme’s effectiveness. The entire fellowship cohort (*n* = 40) was included in this evaluation, and no formal sample size calculation was, therefore, undertaken, with sample size determined by the number of pharmacists enrolled in UK-ALF-A. A pre-post fellowship evaluation design was used.

Demographic analysis was conducted using the information provided in the application forms, including geographical location, gender, age, years of pharmacy practice, and academic qualifications. Demographic data were summarised descriptively using frequencies and percentages. Quantitative assessments were conducted at programme entry (baseline) and completion (12 months), while qualitative data were collected at fellowship completion through surveys and semi-structured interviews.

During the preparation of this manuscript, the authors used ChatGPT (OpenAI, version GPT-5.6) and Microsoft Copilot (version 2607) to assist with language editing, manuscript revision, and quality assurance. These tools were used to improve grammar and readability, revise text in response to author and reviewer comments, identify potential inconsistencies in data analysis reporting, and verify consistency between figures, results, references, and the manuscript narrative. No generative AI tools were used to generate original scientific content, analyse data, interpret results, or draw scientific conclusions. All outputs were critically reviewed, verified, and edited by the authors, who take full responsibility for the accuracy, integrity, and content of the manuscript.

### 4.1. UK-ALF-A Fellowship Programme and Evaluation Timeline

UK-ALF-A was delivered over 12 months (April 2025 to March 2026) and comprised structured learning activities, mentorship, peer learning and workplace-based AMS continuous quality improvement projects. The blended learning programme included online learning modules, live webinars, peer-learning sessions, one residential workshop, and regular mentor meetings. The sequencing of programme activities and evaluation assessments is shown in Table 3.

### 4.2. Fellowship Self-Assessment and 360-Degree Feedback from Colleagues

A fellowship self-assessment and 360-degree feedback were completed both at programme entry and completion using the International Pharmaceutical Federation (FIP) Global Advanced Development Framework (GADF) to assess changes in fellows’ advanced practice competence in AMS. The FIP GADF is an internationally developed, validated and widely recognised professional development framework that provides competency descriptors and progressive stages of advanced pharmacy practice [2].

The FIP GADF maps three broad-based advanced pharmacy practice stages across six clusters, each characterised by specific competencies (Table 4). For the purposes of this evaluation, only competencies within Clusters 1–4 were included, as these were considered most relevant to the fellowship objectives. For each cluster, competencies are defined through descriptors across three progressive stages of advanced pharmacy practice:

- Advanced Stage 1 (AS1) reflects practitioners demonstrating advanced practice beyond routine professional requirements.
- Advanced Stage 2 (AS2) reflects practitioners who influence practice more broadly and contribute to service development and improvement.
- Advanced Stage 3 (AS3) reflects practitioners demonstrating highly advanced practice, including strategic leadership, system-level influence, and organisational or professional impact.

Fellows were asked to self-assess, pre- and post-fellowship, their perceived stage of practice for each of the competencies in clusters 1–4, using the competency descriptors provided by the FIP GADF. In addition, each fellow invited at least five colleagues (supervisors, peers, and direct reports) to provide anonymous 360-degree feedback also at both baseline and programme completion. The same assessment framework was used for both self-assessment and 360-degree feedback, enabling comparison between participants’ self-perceptions and colleague feedback.

For each competency within Clusters 1–4 (total 21 competencies), fellows were assessed against the FIP GADF descriptors and categorised as AS1, AS2 or AS3. Stages were not assigned by the study team. Rather, fellows self-assessed their level of practice using the FIP GADF framework descriptors, while colleagues completing the 360-degree feedback assessed fellows using the same descriptors and competency framework. Aggregated feedback was also provided to fellows to support reflection and development planning.

Since the FIP GADF is an internationally developed and widely recognised professional development framework, no additional psychometric validation was undertaken for this evaluation. Fellows and colleagues used the published FIP GADF [2] competency descriptors and stage definitions to assess practice level consistently across the four selected domains.

Quantitative data from the self-assessments and 360-degree feedback were analysed and compared across the following categories:

- FIP GADF Clusters 1, 2, 3, 4 and Advanced Practice Stages 1, 2 or 3;
- Respondent type: Fellows (self-assessment) or colleagues (360-degree feedback);
- Geographical location: UK-based fellows (*n* = 20) and Africa-based fellows (*n* = 20).

Quantitative data were analysed using Microsoft Excel (version 2607) and jamovi (version 2.7.32.0). Advanced Practice Stage ratings were coded as AS1 = 1, AS2 = 2 and AS3 = 3, with higher scores representing higher levels of advanced practice stage. Although stages were coded numerically for analysis, the underlying stages represent ordered categories. For each fellow, mean scores were calculated across the competencies within each FIP GADF cluster and an overall mean score was calculated across all 21 competencies.

For the 37 fellows with paired pre- and post-fellowship self-assessments, changes in cluster and overall scores were assessed using two-sided Wilcoxon signed-rank tests. Mean and median within-fellow changes were calculated as post-fellowship minus pre-fellowship scores. Effect sizes were expressed as paired rank-biserial correlations (r_rb_), with positive values indicating a predominance of higher post-fellowship scores.

For 360-degree feedback, colleague ratings were first averaged within fellow and competency, and these fellow-level competency scores were subsequently averaged within cluster and across all 21 competencies. An overall advanced practice score was calculated from the 21 competency ratings.

For 360-degree colleague feedback, individual colleague responses were anonymous and could not be linked longitudinally. Colleague ratings were therefore first averaged within fellow and competency, giving each colleague equal weighting within the fellow-level competency score, and then averaged across competencies to generate cluster and overall scores. Fellows with both pre- and post-fellow-level 360-degree data were included in paired analyses (*n* = 36).

Competency-level analyses were considered exploratory. Two-sided Wilcoxon signed-rank tests were performed for each of the 21 competencies, with Benjamini–Hochberg false discovery rate (FDR) correction applied separately to the 21 competency-level comparisons within each respondent group. Statistical significance was defined as *p* < 0.05 for primary analyses and q < 0.05 for FDR-adjusted exploratory competency-level analyses.

### 4.3. Post-Fellowship Feedback Survey

Post-fellowship feedback was collected using an online survey to evaluate perceived impact on leadership development, AMS practice, and integration of the fellowship learning into routine work. The survey was designed specifically for the UK-ALF-A fellowship and incorporated multiple question types (including Likert scales, rating grids, and free-text fields) covering demographics, programme content and tools, individual and organisational impact, and support received during the fellowship.

The post-fellowship survey was developed by the evaluation team specifically for the UK-ALF-A evaluation with content informed by the fellowship objectives and evaluation domains, including leadership development, AMS practice, organisational impact, programme delivery and mentorship. The survey was piloted with members of the programme team and refined to improve question structure, flow, branching logic and estimated completion time. All fellows were invited via email to complete the survey electronically through Google Forms.

Data analysis involved descriptive quantitative analysis of closed-ended responses and thematic analysis of open-ended questions. Free-text responses were reviewed, coded, and grouped into thematic categories based on recurring patterns to inform interpretation of fellows’ experiences and support data triangulation.

### 4.4. Semi-Structured Interviews

Semi-structured interviews were conducted to further explore fellows’ views and experiences of participating in the fellowship. The interview guide comprised open-ended questions and was structured around key domains of interest: personal and professional development (including perceived changes in leadership behaviour and influence on workplace practices; organisational impact; AMS practice; challenges and barriers; mentorship; integration of leadership training into day-to-day work; and suggestions for future programme improvement.

Seven fellows were selected for post-programme interviews using a stratified random sampling approach. Stratification criteria were applied to ensure geographical coverage (UK-based and Africa-based fellows) and gender balance.

Interviews were conducted remotely via a secure videoconferencing platform to accommodate the geographical spread of participants across the UK and Africa. Each interview lasted between 30 and 60 min. Interviews followed the semi-structured format to allow comparability across participants while providing flexibility for probing and exploration of emerging themes. Interviews were audio-recorded with participant permission and transcribed verbatim. Field notes were used to supplement audio-recorded data, capturing contextual observations and non-verbal cues relevant to interpretation.

Transcripts were anonymised and assigned unique identifiers. A thematic analysis approach was used to analyse the data. Initially, transcripts were read repeatedly to develop familiarity, followed by open coding to identify key concepts and recurring ideas. Codes were then grouped into broader themes aligned with the interview guide (e.g., leadership development, organisational impact, mentorship, barriers) while also allowing for inductive theme generation where new insights emerged. Themes were reviewed and refined collaboratively to ensure consistency and credibility. Findings were synthesised and triangulated with survey data to strengthen the robustness of the evaluation.

## 5. Conclusions

This study demonstrates that UK-ALF-A is a feasible and evidence-based model for developing AMS-focused leadership capability among pharmacists across diverse health system contexts. Building on prior scoping work [1] and the previous CPhOGHF and ALF-A fellowships, UK-ALF-A extends the evidence base by embedding leadership development within a bidirectional, cross-context fellowship model. This approach not only addresses unmet leadership development needs identified in LMIC settings but also facilitates reciprocal learning and system-level insight across global contexts.

The findings show that the fellowship was associated with substantial improvements in advanced practice competence, particularly within the FIP GADF domains of Expert Professional Practice and Management. Fellows also reported increased confidence in multidisciplinary engagement, communication with prescribers and colleagues, influencing practice, leading quality improvement initiatives, and contributing to AMS-related organisational activities. These competencies are directly relevant to everyday AMS practice, enabling pharmacists to engage more effectively in prescribing discussions, stewardship improvement activities, and organisational decision-making. Although objective AMS outcomes were not measured, fellows reported applying leadership skills to antimicrobial prescribing discussions, stewardship activities, multidisciplinary working and organisational improvement initiatives. The particularly strong gains observed among Africa-based fellows highlight the role of structured leadership development in addressing global inequities in workforce capability.

Importantly, the study contributes to a growing body of evidence demonstrating that leadership development is not peripheral, but fundamental to AMS capacity building. By linking leadership development with applied service improvement, mentorship and systems thinking, UK-ALF-A provides a practical and evidence-informed model for strengthening AMS through workforce investment.

Despite challenges related to programme intensity, mentoring variability and accessibility, the fellowship was consistently perceived as transformative, with impact extending beyond the programme through sustained networks, enhanced professional credibility and ongoing leadership engagement.

Overall, UK-ALF-A was associated with significant improvements in advanced practice competency and qualitative evidence of enhanced AMS-focused leadership capability. The findings support the value of structured, mentored and bidirectional leadership development as a component of AMS workforce capacity building, while further research is required to determine whether these capability gains translate into measurable downstream AMS outcomes.

## Figures and Tables

**Table 1:** Demographic characteristics of UK-ALF-A participants (*n* = 40).

Characteristic *	UK (*n* = 20)	Africa (*n* = 20)	Total (*n* = 40)
Gender, *n* (%)			
Female	18 (90)	12 (60)	30 (75)
Male	2 (10)	8 (40)	10 (25)
Age group (years), *n* (%)			
21–30	6 (30)	6 (30)	12 (30)
31–40	10 (50)	11 (55)	21 (52.5)
41–50	4 (20)	3 (15)	7 (17.5)
Years in pharmacy practice, *n* (%)			
1–5	4 (20)	7 (35)	11 (27.5)
6–10	7 (35)	6 (30)	13 (32.5)
11–15	7 (35)	6 (30)	13 (32.5)
Not specified	2 (10)	1 (5)	3 (7.5)
Highest academic qualification, *n* (%)			
Bachelor’s degree, e.g., BPharm/BSc	0 (0)	15 (75)	15 (37.5)
Master of Pharmacy (MPharm)	20 (100)	2 (10)	22 (55)
Master’s Degree, e.g., MSc	0 (0)	2 (10)	2 (5)
Doctor of Pharmacy (PharmD)	0 (0)	1 (5)	1 (2.5)

* Data are presented as *n* (%). Demographic information was obtained from fellowship application forms completed prior to enrolment in the fellowship. Percentages are calculated within each geographical group (UK and Africa) and for the overall cohort. The evaluation included the entire fellowship cohort (*n* = 40); therefore, demographic data are presented descriptively only.

**Table 2:** Changes in FIP GADF competency scores following UK-ALF-A. Self-assessment (by fellows, *n* = 37 paired responses) and 360-degree-feedback (by colleagues, *n* = 36 paired responses) showed a significant improvement across all four FIP GADF clusters (Wilcoxon signed-rank *p* < 0.001 across all clusters).

FIP GADF Domain	Self-Assessment Median/Mean Change	360-Degree Feedback Median/Mean Change
1. Expert Professional Practice	+0.75/+0.70	+0.53/+0.53
2. Working with Others	+0.50/+0.47	+0.55/+0.56
3. Leadership	+0.67/+0.60	+0.58/+0.54
4. Management	+0.56/+0.56	+0.49/+0.57
Overall score (Clusters 1–4)	+0.57/+0.59	+0.55/+0.55

**Table 3:** UK-ALF-A fellowship and evaluation activity timeline.

Timing	Fellowship Stage	Evaluation Activities
March 2025	Fellow selection and onboarding	- Demographic data collection and analysis
April 2025	Fellowship commencement	- Pre-fellowship self-assessment and 360-degree feedback
March 2026	Fellowship completion	- Post-fellowship self-assessment and 360-degree feedback - Post-fellowship survey - Post-fellowship semi-structured interviews

**Table 4:** Advanced pharmacy practice stages and clusters of the International Pharmaceutical Federation (FIP) Global Advanced Development Framework (GADF). Fellows completed a self-assessment, and colleagues completed 360-degree feedback using the competency descriptors provided within the framework.

FIP GADF Cluster	No. of Competencies	Advanced Pharmacy Practice Stages *
1. Expert Professional Practice	4	AS1, AS2, AS3
2. Working with Others	2	AS1, AS2, AS3
3. Leadership	6	AS1, AS2, AS3
4. Management	9	AS1, AS2, AS3
5. Education, Training and Development	Not used in this evaluation	
6. Research and Evaluation	Not used in this evaluation	

* Abbreviations: AS1, Advanced Stage 1; AS2, Advanced Stage 2; AS3, Advanced Stage 3. The stages represent progressively higher levels of advanced pharmacy practice as defined by the FIP GADF.

## Data Availability

Data supporting the findings of this study are not publicly available due to participant confidentiality and ethical restrictions. De-identified datasets may be available from the corresponding authors upon reasonable request and subject to approval from the programme governance team and data protection requirements.

## Supplementary Materials

The following supporting information can be downloaded at: https://www.mdpi.com/article/10.3390/antibiotics15090833/s1. Primary Evaluation Summary. Table S1: Baseline characteristics of the assessment dataset of pre- and post-fellowship scores across FIP GADF competency clusters. Table S2: Summary of pre- and post-fellowship scores across FIP GADF competency clusters and overall composite score for fellow self-assessments (*n* = 37 paired) and 360-degree colleague feedback (*n* = 36 paired fellow codes). Table S3: Item-level statistical analysis across all 21 FIP GADF competencies for fellow self-assessments (*n* = 37) and 360-degree colleague feedback (*n* = 36), including Benjamini–Hochberg False Discovery Rate (FDR) adjusted *p*-values. All 21 competencies were statistically significantly higher post-fellowship in both the paired self-assessment analysis and the matched fellow-code 360-degree analysis after FDR adjustment. Secondary Evaluation Summary. Table S4: Exploratory comparison of pre-fellowship, post-fellowship, and mean score changes between UK-based fellows and Africa-based fellows across self-assessment clusters.
