# Supplementary material for: UK–Africa Leadership Fellowship for Antimicrobial Stewardship (UK-ALF-A): A Bilateral Learning Approach to Advancing AMS Expertise Among Pharmacists
Source: Antibiotics (Basel). 2026 Aug 27;15(9):833. doi: 10.3390/antibiotics15090833 (PMC13603237; doi:10.3390/antibiotics15090833)
Supplement: Supplementary file 1 [file antibiotics-15-00833-s001.zip › antibiotics-4475591-supplementary.pdf]

Supplementary data

UK-Africa Leadership Fellowship for Antimicrobial Stewardship (UK-ALF-A): A Bilateral Learning Approach to Advancing AMS Expertise Among Pharmacists

Siân Price, Yen Truong, Paria Sanaty Zadeh, Gloria Tumukunde, Claire Brandish, Edwin Panford-Quainoo, Sarah Cavanagh, Esther Obiri-Yeboah, Maxencia Nabiryo, Elizabeth Ward, Gizem Gülpinar, Victoria Rutter, Helena Rosado*, Chikondi Savieli*

Primary Evaluation Summary

Table S1: Baseline characteristics of the assessment dataset of pre- and post-fellowship scores across FIP GADF competency clusters.

Assessment source	Timepoint	Assessment records available	Overall FIP GADF stage score	
			Mean (SD)	Median (IQR)
Fellow self-assessment	Pre	40 fellow responses	1.65 (0.46)	1.52 (0.73)
Fellow self-assessment	Post	37 fellow responses	2.28 (0.44)	2.33 (0.57)
360° colleague feedback	Pre	173 colleague responses	1.81 (0.52)	1.81 (0.81)
360° colleague feedback	Post	115 colleague responses	2.38 (0.52)	2.48 (0.79)

Table S2: Summary of pre- and post-fellowship scores across FIP GADF competency clusters and overall composite score for fellow self-assessments (n=37 paired) and 360-degree colleague feedback (n=36 paired fellow codes).

Evaluation Group / Cluster Domain	Pre-Mean (SD)	Post-Mean (SD)	Pre-Median (IQR)	Post-Median (IQR)	Mean Change	Median Change	Wilcoxon p-value	Rank-Biserial (r_{rb})
**Fellow Self-Assessment** (n=37)								
1: Expert Professional Practice	1.77 (0.46)	2.47 (0.38)	1.75 (0.75)	2.50 (0.50)	+0.70	+0.75	< 0.001	0.97
2: Working with Others	1.93 (0.60)	2.41 (0.47)	2.00 (1.00)	2.50 (1.00)	+0.47	+0.50	< 0.001	0.86
3: Leadership	1.70 (0.53)	2.30 (0.47)	1.67 (0.67)	2.33 (0.67)	+0.59	+0.67	< 0.001	0.90
4: Management	1.60 (0.45)	2.17 (0.50)	1.67 (0.67)	2.22 (0.56)	+0.56	+0.56	< 0.001	0.86
Overall (Cluster 1-4)	***1.69 (0.45)***	***2.28 (0.44)***	***1.67 (0.81)***	***2.33 (0.57)***	***+0.59***	***+0.57***	***< 0.001***	***0.90***
**360-Degree Colleague Ratings** (n=36)								
1: Expert Professional Practice	1.93 (0.45)	2.46 (0.41)	1.91 (0.64)	2.52 (0.52)	+0.53	+0.53	< 0.001	0.89
2: Working with Others	1.95 (0.42)	2.51 (0.42)	2.00 (0.54)	2.50 (0.66)	+0.56	+0.55	< 0.001	0.90
3: Leadership	1.83 (0.44)	2.36 (0.45)	1.80 (0.58)	2.45 (0.61)	+0.54	+0.58	< 0.001	0.89
4: Management	1.73 (0.42)	2.31 (0.42)	1.62 (0.54)	2.30 (0.62)	+0.57	+0.49	< 0.001	0.93
Overall (Cluster 1-4)	***1.82 (0.41)***	***2.37 (0.41)***	***1.78 (0.54)***	***2.43 (0.57)***	***+0.55***	***+0.55***	***< 0.001***	***0.90***

Table S3: Item-level statistical analysis across all 21 FIP GADF competencies for fellow self-assessments (n=37) and 360-degree colleague feedback (n=36), including Benjamini–Hochberg False Discovery Rate (FDR) adjusted p-values. All 21 competencies were statistically significantly higher post-fellowship in both the paired self-assessment analysis and the matched fellow-code 360-degree analysis after FDR adjustment.

Competency	Pre-Mean	Post-Mean	Mean-Change	Raw p-value	FDR q-value	r_{rb}
**Fellow Self-Assessment** (n=37)						
1.1 Expert skills and knowledge	1.57	2.27	+0.70	< 0.001	< 0.001	0.80
1.2 Developing professional expertise	1.70	2.51	+0.81	< 0.001	< 0.001	0.93
1.3 Reasoning and judgement	1.78	2.57	+0.78	< 0.001	< 0.001	0.93
1.4 Professional autonomy	2.03	2.51	+0.49	0.0015	0.0020	0.63
2.1 Communication	1.86	2.30	+0.43	0.0017	0.0020	0.66
2.2 Teamwork and consultation	2.00	2.51	+0.51	0.0014	0.0020	0.73
3.1 Strategic context	1.76	2.30	+0.54	< 0.001	< 0.001	0.81
3.2 Governance	1.59	2.05	+0.46	0.0017	0.0020	0.72
3.3 Vision	1.59	2.24	+0.65	< 0.001	< 0.001	0.71
3.4 Innovation	1.59	2.35	+0.76	< 0.001	< 0.001	0.86
3.5 Service development	1.89	2.38	+0.49	0.0019	0.0021	0.78
3.6 Motivational leadership	1.78	2.46	+0.68	< 0.001	< 0.001	1.00
4.1 Responding and adapting to national needs	1.57	2.05	+0.49	< 0.001	< 0.001	0.81
4.2 Resource utilisation	1.78	2.30	+0.51	< 0.001	< 0.001	0.81
4.3 Standards of practice	1.65	2.14	+0.49	0.0057	0.0057	0.55
4.4 Managing risk	1.35	1.81	+0.46	0.0017	0.0020	0.72
4.5 Managing performance	1.70	2.14	+0.43	0.0026	0.0027	0.70
4.6 Project management	1.62	2.22	+0.59	< 0.001	< 0.001	1.00
4.7 Managing change	1.62	2.35	+0.73	< 0.001	< 0.001	0.80
4.8 Strategic planning	1.43	2.14	+0.70	< 0.001	< 0.001	0.92
4.9 Working across boundaries	1.70	2.35	+0.65	< 0.001	< 0.001	0.73
**360-Degree Colleague Ratings** (n=36)						
1.1 Expert skills and knowledge	1.80	2.37	+0.57	< 0.001	< 0.001	0.90
1.2 Developing professional expertise	1.95	2.38	+0.43	< 0.001	< 0.001	0.83
1.3 Reasoning and judgement	1.98	2.54	+0.56	< 0.001	< 0.001	0.82
1.4 Professional autonomy	2.00	2.55	+0.54	< 0.001	< 0.001	0.84
2.1 Communication	1.86	2.48	+0.61	< 0.001	< 0.001	0.91
2.2 Teamwork and consultation	2.03	2.54	+0.51	< 0.001	< 0.001	0.84
3.1 Strategic context	1.83	2.39	+0.56	< 0.001	< 0.001	0.79

3.2 Governance	1.75	2.27	+0.51	< 0.001	< 0.001	0.89
3.3 Vision	1.69	2.28	+0.58	< 0.001	< 0.001	0.81
3.4 Innovation	1.84	2.39	+0.54	< 0.001	< 0.001	0.82
3.5 Service development	1.95	2.46	+0.51	< 0.001	< 0.001	0.87
3.6 Motivational leadership	1.89	2.39	+0.50	< 0.001	< 0.001	0.82
4.1 Responding and adapting to national needs	1.65	2.10	+0.45	< 0.001	< 0.001	0.70
4.2 Resource utilisation	1.95	2.42	+0.47	< 0.001	< 0.001	0.82
4.3 Standards of practice	1.77	2.34	+0.57	< 0.001	< 0.001	0.96
4.4 Managing risk	1.56	2.16	+0.60	< 0.001	< 0.001	0.84
4.5 Managing performance	1.70	2.36	+0.66	< 0.001	< 0.001	0.92
4.6 Project management	1.73	2.34	+0.61	< 0.001	< 0.001	0.85
4.7 Managing change	1.71	2.39	+0.68	< 0.001	< 0.001	0.98
4.8 Strategic planning	1.69	2.23	+0.53	< 0.001	< 0.001	0.89
4.9 Working across boundaries	1.84	2.45	+0.61	< 0.001	< 0.001	0.85

Secondary Evaluation Summary

Among the 37 paired fellows, the magnitude of self-reported improvement was larger in Africa-based fellows than UK-based fellows across all four clusters. This should be regarded as exploratory because the sample is small, baseline competency differed between regions (higher baseline for UK-based fellows compared to Africa-based fellows), and the study was not designed or powered primarily to test regional interaction.

Table S4: Exploratory comparison of pre-fellowship, post-fellowship, and mean score changes between UK-based fellows and Africa-based fellows across self-assessment clusters.

Competency Cluster	**UK** (n=19)			**Africa** (n=18)		
	**Pre-Mean**	**Post-Mean**	**Mean Change**	**Pre-Mean**	**Post-Mean**	**Mean Change**
**Cluster 1: Expert Practice**	1.97	2.45	+0.47	1.56	2.49	+0.93
**Cluster 2: Working with Others**	2.05	2.34	+0.29	1.81	2.47	+0.67
**Cluster 3: Leadership**	1.87	2.23	+0.36	1.53	2.37	+0.84
**Cluster 4: Management**	1.77	2.06	+0.29	1.43	2.27	+0.85
**Overall (Cluster 1-4: 21 Competencies)**	1.86	2.21	+0.35	1.52	2.36	+0.84
